# Plasmapheresis as a Life-Saving Measure for Severe Hypertriglyceridemia-Induced Acute Pancreatitis

**DOI:** 10.7759/cureus.94451

**Published:** 2025-10-13

**Authors:** Fahad Neduvancheri, Fathima Shahana Sumi, Rooby Shaheer, Muhammed Shereef M, Sajeesh Sivadas, Thejus Kallarikkandi, Ranjit Narayanan

**Affiliations:** 1 Gastroenterology, Malabar Institute of Medical Sciences Ltd., Kottakkal, IND; 2 Nephrology, Malabar Institute of Medical Sciences Ltd., Kottakkal, IND; 3 Surgery, Malabar Institute of Medical Sciences Ltd., Kottakkal, IND; 4 Critical Care, Malabar Institute of Medical Sciences Ltd., Kottakkal, IND

**Keywords:** acute pancreatitis, diabetic ketoacidosis, hypertriglyceridemia, plasmapheresis, respiratory failure

## Abstract

Severe hypertriglyceridemia (HTG) presenting as acute pancreatitis (AP) in the setting of diabetic ketoacidosis (DKA) is a rare combination, reported as the enigmatic triad in literature. Here we present a case of a 26-year-old woman, with diabetes mellitus (DM) for three years, who presented with features suggestive of acute pancreatitis complicated with DKA in addition to systemic inflammatory response syndrome (SIRS) and acute respiratory distress syndrome (ARDS). Evaluation revealed severe HTG to the tune of 9947 mg/dL. She was managed with insulin infusion combined with three sessions of plasmapheresis, which led to marked clinical and biochemical improvement. This case highlights the importance of plasmapheresis as a lifesaving therapeutic option for severe HTG-induced AP.

## Introduction

The majority of acute pancreatitis (AP) is caused by gallstones (40%-70%) and alcohol (25%-35%) [1]. Hypertriglyceridemia may be associated with AP, usually when their levels are higher than 1000 mg/dL [2,3]. We report a case of a 26-year-old woman with hypertriglyceridemia and uncontrolled diabetes who presented to the emergency room (ER) with a clinical diagnosis of AP and, on evaluation, had diabetic ketoacidosis (DKA) and respiratory dysfunction.

A marked improvement in the patient's general condition and laboratory parameters was noticed following plasmapheresis. This case report highlights the role of plasmapheresis in the rapid reduction of severe hypertriglyceridemia in the setting of AP. In this instance, it served as a lifesaving therapeutic modality.

## Case presentation

A 26-year-old woman with diabetes mellitus for three years and treatment non-compliance, presented to our ER with severe epigastric pain and multiple episodes of vomiting suggestive of acute pancreatitis. She denied any history of alcohol use. The patient was afebrile, with a blood pressure of 120/70 mmHg, heart rate of 92/min, and oxygen saturation of 97% on room air. Initial blood workup (Table 1) showed leukocytosis (17570/μL) with elevated C-reactive protein (297 mg/dL) and high serum lipase (13671 IU/L) and amylase (313 IU/L) levels. Her liver function tests (LFTs) and renal function tests (RFTs) were within normal limits. Blood sugars were 229 mg/dL at the time of presentation with a HbA1c of 12.9% indicating poorly controlled DM. Computed tomography of the abdomen showed bulky pancreas with peripancreatic fat stranding with no areas of calcification or evidence of gallstones--suggestive of acute pancreatitis. According to the Modified Marshall Score [4], she was initially managed conservatively with bowel rest and intravenous Ringer’s lactate. However, she developed acute onset dyspnea with desaturation from the ward approximately four hours after admission, necessitating shift to the medical intensive care unit.

**Table 1:** Initial blood investigation reports.

Lab test	Result	Reference value
Hemoglobin	20.0 g/dL	12-15
Total leukocyte count	17570/μL	4000-10000
Platelet	4.18 lakhs/μL	1.5-4.10
Amylase	313 IU/ L	30-110
Lipase	13671 IU/L	23-300
Creatinine	0.6 mg/dL	0.5-1.0
Blood urea nitrogen	6.14 mg/dL	9-20
Random blood sugar	229	<200
Total bilirubin	0.5	0.2-1.3
Direct bilirubin	0.3	0.0-0.3
Alanine transaminase	33 U/L	14-36
Aspartate transaminase	18 U/L	14-36
Alkaline phosphatase	117 U/L	38-126
Sodium	134 mmol/L	137-145
Potassium	5.4 mmol/ L	3.5-5.1
Calcium (corrected)	8.8 mg/dL	8.4-10.2
Triglycerides	9947 mg/dL	<150

Further workup revealed high glucose levels (503 mg/dL) and high-anion-gap metabolic acidosis (Table 2), with 4+ blood ketones. Etiological workup for acute pancreatitis revealed severe hypertriglyceridemia (9947 mg/dL) with normal corrected calcium levels (8.8 mg/dL).

**Table 2:** Arterial blood gas analysis on day one of ICU (on 6 L oxygen per minute). PaO₂: partial pressure of oxygen; PaCO₂: partial pressure of carbon dioxide; HCO₃: bicarbonate.

ABG	Patient value	Reference range
pH	6.95	7.35-7.45
PaO_2_	139 mmHg	180-220
PaCO_2_	14 mmHg	35-45
HCO_3_	3.1 mmol/L	22-26
Lactate	2.9 mmol/L	0.5-2.2
Anion gap	19.2 mmol/L	8-14

**Figure 1:**
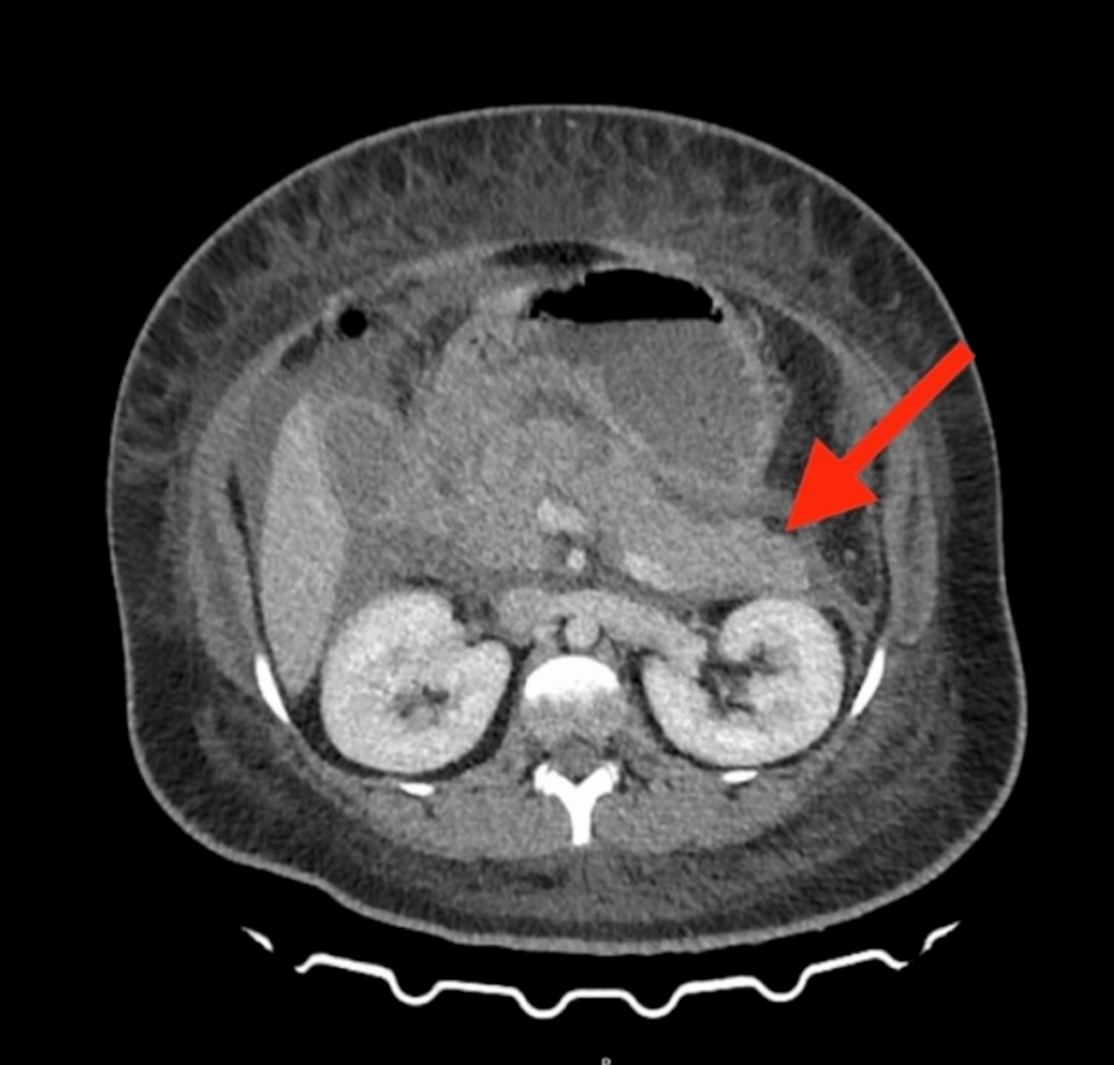
CT scan of abdomen and pelvis with contrast showing bulky pancreas with peripancreatic fat stranding and non-enhancing necrotic areas seen. Peripancreatic fluid noted extending to anterior renal fascia, para-colic gutters and forming a collection in pelvis.

She was promptly started on noninvasive ventilation (NIV), insulin infusion, and intensive intravenous fluid therapy (at the rate of 1.5 mL/kg/h). Subsequently, a contrast-enhanced computed tomography of the abdomen showed diffusely bulky pancreas with peripancreatic fat stranding and non-enhancing necrotic areas (Figure 1). Peripancreatic fluid was noted extending to the pelvis. The modified CT severity index was 8, suggesting severe involvement.

**Figure 2:**
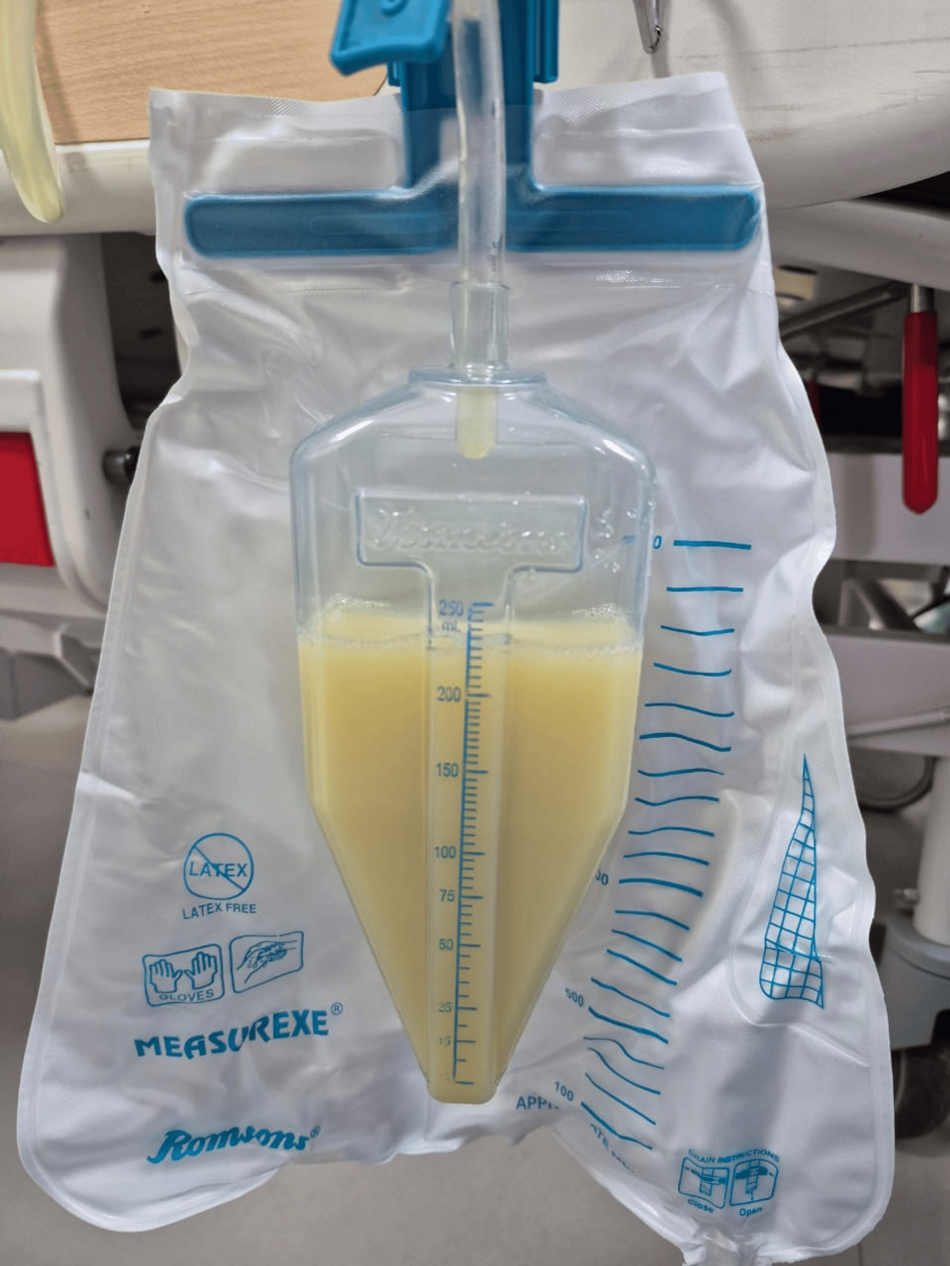
Chylous plasma filtered by plasmapheresis.

**Figure 3:**
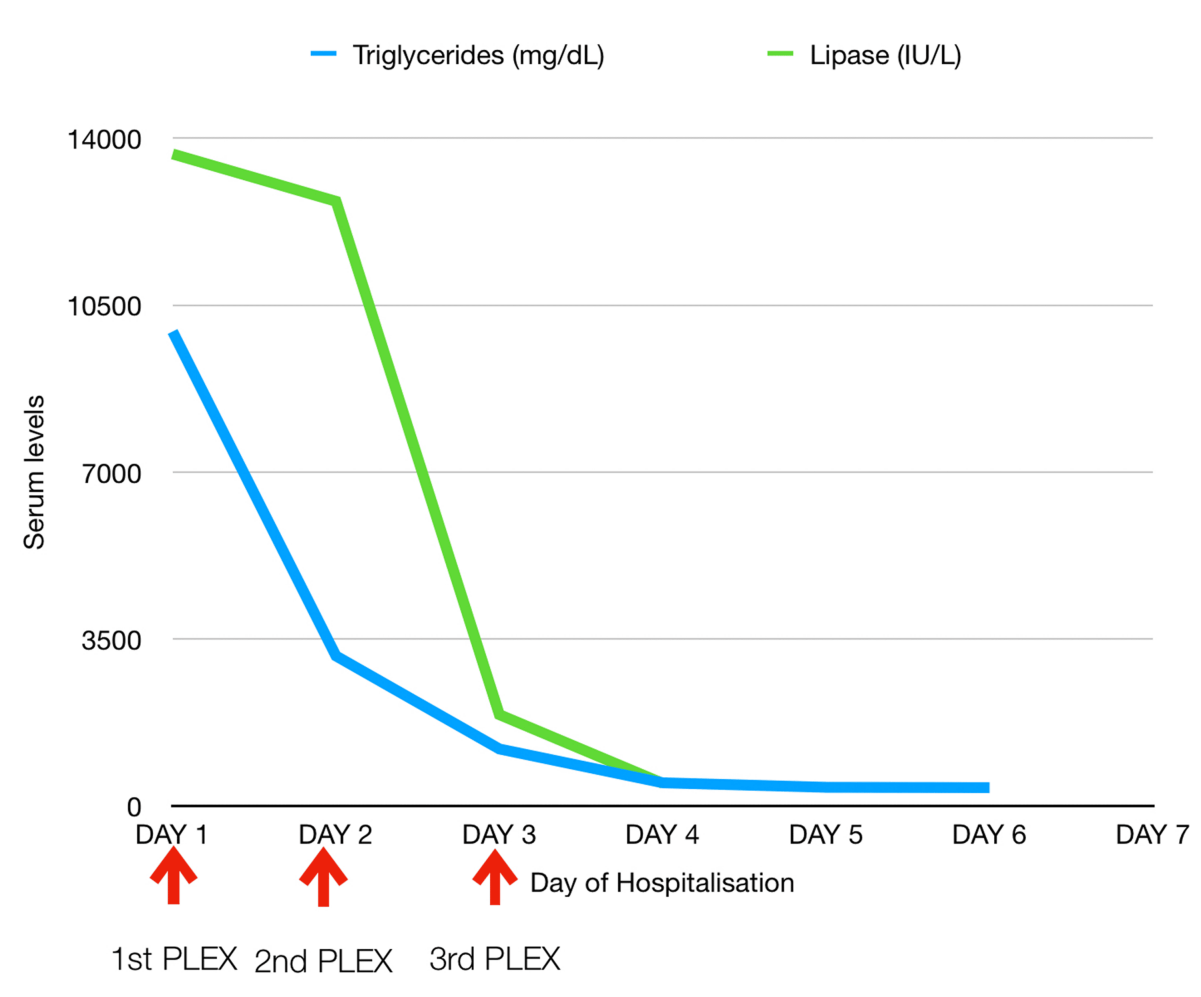
Decreasing trend of triglyceride and lipase levels after initiating plasmapheresis (PLEX).

Considering the drastic worsening of the patient's general condition and high levels of triglycerides, a decision to undertake plasmapheresis was made in consultation with nephrology. A total of 1.75 liters of plasma was removed (Figure 2) and replaced with fresh frozen plasma (FFP) and 5% albumin. Following the first session of plasmapheresis, her triglyceride levels dropped down to 3144 mg/dL. After the second session, it decreased to 1193 mg/dL, and after the third session, it decreased to 484 mg/dL (Figure 3). In total, three sessions of plasmapheresis were performed. The patient improved clinically and was shifted out of the intensive care unit by day four. Her subsequent hospital course was complicated by persistent fever with negative blood and urine cultures. She improved in due course with broad-spectrum empirical antibiotics (meropenem) and was discharged on day 12 in a clinically stable condition.

## Discussion

The triad of DKA, hypertriglyceridemia, and acute pancreatitis is a rare clinical occurrence, reported in only 4% of cases [5]. The bidirectional relationship between uncontrolled diabetes and hypertriglyceridemia has been highlighted previously [6]. While uncontrolled diabetes is a well-documented cause of hypertriglyceridemia, triglycerides as high as 9000 mg/dL suggests a primary etiology [7].

The role of plasmapheresis in HTG-induced AP still remains controversial, with literature presenting two contrasting perspectives. While specific therapies like heparin and insulin infusion are indicated in hypertriglyceridemia-induced acute pancreatitis [8], the American Society of Apheresis guidelines have approved the use of therapeutic plasmapheresis in the setting of worsening systemic inflammation or lactic acidosis [9].

In a study involving 111 episodes of HTG-induced AP treated with plasmapheresis, the average reduction in triglycerides following plasma exchange was 59%, which was double the reduction seen with conservative treatment (27%) [10]. Absolute indications for plasmapheresis, as cited in previous literature, include (a) pancreatitis refractory to pharmacological approaches, (b) serum triglycerides >1000 mg/dL, (c) serum lipase exceeding three times the upper limit of normal, (d) hypocalcemia, (e) lactic acidosis, and (f) worsening inflammation and organ dysfunction [11]. As our case fulfilled five out of the six criteria mentioned, plasmapheresis was deemed the preferred therapeutic option.

Although there has been no evidence of mortality advantage with plasmapheresis, a study focusing on 25 patients with triglyceride levels exceeding 5000 mg/dL found that the patients who received plasmapheresis had a significantly shorter hospital stay of five days compared to 11 days in those who did not receive it [12]. It has also been reported that plasma exchange improves outcomes by removing pro-inflammatory markers and cytokines, which further dampens the inflammatory process in HTG-induced AP [7]. Evidence also suggests that the early initiation of plasmapheresis, that is, within 24 to 96 hours after the onset of symptoms, has proven beneficial [13].

## Conclusions

This case reinforces the value of considering plasmapheresis as a therapeutic option in carefully selected patients with hypertriglyceridemia-induced acute pancreatitis with serious sequelae. Our experience highlights that timely initiation may help in stabilizing patients with high-risk features such as significant organ dysfunction and extreme triglyceride levels. Additionally, the favorable outcome in our patient underscores the importance of multidisciplinary decision-making and individualized treatment strategies in critical care settings, while also recognizing that recent evidence has questioned its routine use because of cost and potential complications. 
